# A microRNA network regulates proliferative timing and extracellular matrix synthesis during cellular quiescence in fibroblasts

**DOI:** 10.1186/gb-2012-13-12-r121

**Published:** 2012-12-22

**Authors:** Eric J Suh, Matthew Y Remillard, Aster Legesse-Miller, Elizabeth L Johnson, Johanna MS Lemons, Talia R Chapman, Joshua J Forman, Mina Kojima, Eric S Silberman, Hilary A Coller

**Affiliations:** 1 Princeton University, Department of Molecular Biology, 14 Washington Rd, Princeton, NJ 08544 USA

**Keywords:** MicroRNA, Quiescence, Cell cycle, Proliferation, Extracellular matrix, Fibroblast, Microarray, miR-29

## Abstract

**Background:**

Although quiescence (reversible cell cycle arrest) is a key part in the life history and fate of many mammalian cell types, the mechanisms of gene regulation in quiescent cells are poorly understood. We sought to clarify the role of microRNAs as regulators of the cellular functions of quiescent human fibroblasts.

**Results:**

Using microarrays, we discovered that the expression of the majority of profiled microRNAs differed between proliferating and quiescent fibroblasts. Fibroblasts induced into quiescence by contact inhibition or serum starvation had similar microRNA profiles, indicating common changes induced by distinct quiescence signals. By analyzing the gene expression patterns of microRNA target genes with quiescence, we discovered a strong regulatory function for *miR-29*, which is downregulated with quiescence. Using microarrays and immunoblotting, we confirmed that *miR-29 *targets genes encoding collagen and other extracellular matrix proteins and that those target genes are induced in quiescence. In addition, overexpression of *miR-29 *resulted in more rapid cell cycle re-entry from quiescence. We also found that *let-7 *and *miR-125 *were upregulated in quiescent cells. Overexpression of either one alone resulted in slower cell cycle re-entry from quiescence, while the combination of both together slowed cell cycle re-entry even further.

**Conclusions:**

microRNAs regulate key aspects of fibroblast quiescence including the proliferative state of the cells as well as their gene expression profiles, in particular, the induction of extracellular matrix proteins in quiescent fibroblasts.

## Background

When mammalian cells are in an environment unfavorable for continued proliferation, they can exit the cell cycle in early to mid-G_1 _phase at the 'restriction point' [1] and enter a reversible, out-of-cell cycle state denoted 'quiescence'. Many cells in the human body are quiescent, and the ability of cells to exit the cell cycle but retain their capacity to re-enter the cell cycle as needed (for instance, when required to replenish a cell lineage, mount an immune response, or heal a wound) is central to normal physiology. Failures in this process may underlie a wide range of pathologies including excessive scarring, fibrotic disease, chronic wounding, and cancer, yet we have a poor understanding of the changes that occur when cells become quiescent or the molecular basis for these changes.

Widespread gene expression changes occur when cells enter quiescence, including both repression and activation of genes [2-9]. These changes can vary among cell types and in response to different antiproliferative signals, but there are also commonalities in different types of quiescence and in different quiescent cell types [2,7-9]. Several important regulators of the gene expression changes that occur with quiescence have been described, including the *MYC *and *E2F *family transcription factors that coordinate cell cycle re-entry and repress cell cycle genes during quiescence [5,10-13], and the *HES1 *transcriptional repressor that preserves the reversibility of quiescence [14]. There are also hundreds of genes that are upregulated when cells become quiescent, whose possible regulators include forkhead transcription factors [15,16], *ELK1*, *NF-κB*, *MEF2*, *IRF*, *AP-1*, *SALL2*, and *MXI1 *[5]. Despite these proposed factors, however, the drivers and mechanisms of many of the gene expression changes in quiescence are still not known.

In addition to regulation of quiescence by transcription factors, there is likely also regulation of quiescence gene expression changes at the post-transcriptional level. microRNAs are 20 to 23 nucleotide non-coding RNAs that regulate a wide variety of transcripts post-transcriptionally by inducing transcript degradation or inhibiting protein translation [17-19]. microRNAs have been implicated in a wide range of biological processes related to quiescence, including cell proliferation control, stem cell renewal, developmental timing, and cancer [20]. Medina and colleagues, for example, discovered that four microRNAs were upregulated and over 100 microRNAs were downregulated as T98G glioblastoma cells progress from quiescence into the proliferative cell cycle [21]. They and others demonstrated that *miR-221 *and *miR-222 *target the cyclin-dependent kinase inhibitors p27^Kip1 ^and p57^Kip2^, such that overexpression of *miR-221 *and *miR-222 *during growth factor deprivation induces S-phase entry and triggers cell death [21-25].

Another example is the *let-7 *family of microRNAs, members of which are important regulators of cellular differentiation [26-34] and proliferation [29,35-37] in mammals, *C. elegans*, and *Drosophila melanogaster*. *let-7 *family members can behave as tumor suppressors and antagonize oncogenes such as *MYC *and *RAS *[28,35,38-45].

As a final example, the *miR-17-92 *cluster of six microRNAs, which is induced by the *MYC *oncogene [46], can itself act as an oncogene. Enforced expression of the *miR-17-92 *cluster, in concert with *MYC *expression, can accelerate tumor development in a mouse B-cell lymphoma model [47]. While *MYC *can induce transcription of *E2F *transcription factors, two of the members of the microRNA cluster, *miR-17-5p *and *miR-20a*, negatively regulate levels of *E2F1*, demonstrating a complex network of interactions that may affect the cell's commitment to proliferation or apoptosis [46-51].

We investigated the role of microRNAs in a fibroblast model of quiescence and discovered that microRNA expression is broadly and similarly altered by two different quiescence signals: contact inhibition and serum withdrawal. We further found that microRNAs regulate some of the changes in gene expression and cellular function associated with quiescence, as well as the transition between proliferation and quiescence.

## Results

### microRNAs exhibit a strong quiescence signature

**Figure 1:**
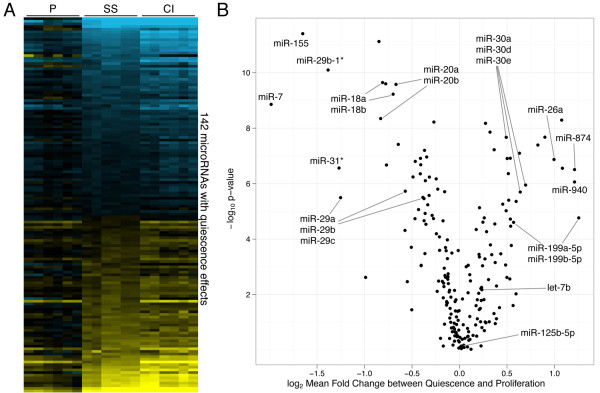
**Widespread changes in microRNA abundance with quiescence**. (**A**) The log_2 _fold-change in the expression of the 142 microRNAs that change expression at a 1% FDR during serum starvation (SS) or contact inhibition (CI) are depicted with respect to their average expression in proliferating (P) cells. Blue and yellow indicate negative and positive values, respectively. Genes are in order of the magnitude of their mean log_2 _fold change from proliferation to quiescence. (**B**) 'Volcano' plot of microRNA average log_2 _fold-change in quiescence conditions on the x-axis *versus *the log_10 _*P *value for the significance of the quiescence parameter in gene expression on the y-axis.

We have developed a model system of quiescence in primary human fibroblasts in which quiescence can be induced by either serum starvation or contact inhibition. Either condition results in an accumulation of quiescent cells, as indicated by cell cycle markers and RNA content [52]. Using one-color microRNA microarrays, we monitored microRNA expression levels in proliferating, serum-starved, and contact-inhibited primary human dermal fibroblasts (Figure 1A). Among the 209 microRNAs detected above background, 142 (68%) were expressed at different levels in proliferating compared with either serum-starved or contact-inhibited fibroblasts at a false-discovery rate of 1% (Figure 1B). microRNA expression patterns for contact inhibition and serum starvation were extremely similar, with a 95% confidence interval (CI) Pearson's correlation of 0.952 to 0.975, much more so than the mRNA expression patterns for the same conditions (Pearson's correlation of 0.319 to 0.341, 95% CI) (Additional File 1, Figure S1). This large difference in the amount of correlation between quiescence states may be due to experimental design or microarray platform differences, but an alternative explanation is that microRNAs exhibit more of a common quiescence signature than protein-coding transcripts [2]. microRNAs downregulated in quiescent cells included *miR-18*, *miR-20*, *miR-29*, and *miR-7*, and microRNAs upregulated with quiescence included *let-7b*, *miR-125a*, *miR-30*, *miR-181*, *miR-26*, and *miR-199*. With a stringent cutoff of greater than two-fold expression change due to quiescence, eight microRNAs were expressed at higher levels in proliferating cells and eight were expressed at higher levels in quiescent cells (Additional File 1, Table S1).

We sought to validate the changes in microRNA levels with an independent method. In collaboration with Rosetta Inpharmatics, we used massively parallel, multiplexed qRT-PCR [53] to monitor the abundance of 219 microRNAs in fibroblasts collected during proliferation or after 4 days of serum starvation. There was strong agreement between the fold-change values obtained via the microarray and the multiplex qRT-PCR (Pearson's correlation 0.504 to 0.751, 95% CI) (Additional File 1, Figure S2).

### Targets of microRNAs change with quiescence

**Figure 2:**
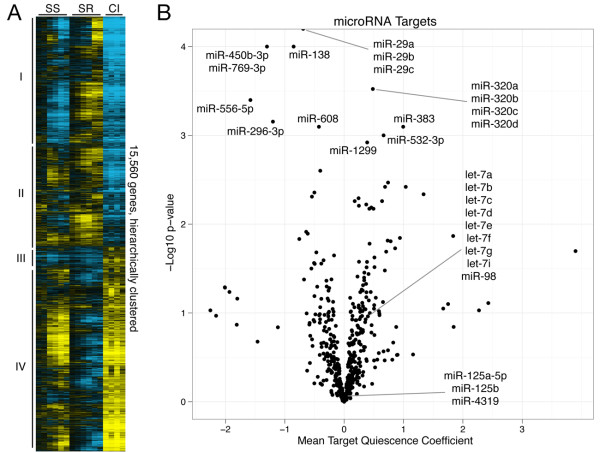
**Changes in target genes with quiescence**. (**A**) Hierarchical clustered heat map representing the log_2 _fold change of gene expression for all 15,560 consistently detectable genes during 1, 2, 4, 8, 24, and 96 h of serum starvation (SS), 1, 2, 4, 8, 24, and 48 h serum restimulation (SR), and 7 and 14 days (each repeated twice) of contact inhibition (CI). Expression in serum starvation and contact inhibition is shown relative to proliferating cells, and expression during serum restimulation is shown relative to 4-day serum-starved cells. Colors are as in Figure 1A. Numerals designate 4 different clusters chosen from the hierarchical clustering tree. Select enriched gene ontology terms for each of the clusters are shown in Additional File 1, Table S2. (**B**) Volcano plot of the mean projection of the microRNA target genes' log_2 _expression onto the array's first eigengene (Additional File 1, Figure S3B) on the x-axis *versus *the log_10 _*P *value of the mean projection on the y-axis.

In order to identify microRNAs with a functional, regulatory role in quiescence, we analyzed the gene expression patterns of microRNA target genes in two whole-genome mRNA microarray timecourses comparing proliferating cells to cells induced into quiescence by contact inhibition or serum starvation (Figure 2A). In one timecourse, fibroblasts were made quiescent by serum withdrawal for 4 days and then re-stimulated with serum for 48 h [54]. In another, fibroblasts were sampled after 7 or 14 days of contact inhibition [52]. Using singular value decomposition of the combined timecourses, we found that the strongest orthonormal gene expression pattern ('eigengene') correlated with the proliferative state of the cell (Additional File 1, Figure S3B). This eigengene explained approximately 40% of the gene expression variation (Additional File 1, Figure S3A). The linear projection of each gene to that eigengene gave a 'proliferation index' for each gene that summarized its association with proliferation or quiescence. For each microRNA, we averaged the proliferation indexes of its predicted target genes as provided by the TargetScan algorithm [55,56] and assigned a *P *value to that mean using bootstrap resampling (Figure 2B). The *miR-29 *family's targets had the most statistically extreme mean proliferation index, with a *P *value <10^-4 ^(the lowest *P *value possible based on the 10^4 ^bootstrap resamplings taken). *miR-29 *expression is strongly associated with proliferation (Additional File 1, Figure S4), and its predicted targets are upregulated by both methods of quiescence induction.

Besides *miR-29*, however, there were few microRNAs with strongly anti-correlated target genes. There are multiple possible explanations. First, expression levels and activity need not be completely correlated, as microRNA activity can be affected by the cooperation or antagonism of RNA-binding proteins [57-60] as well as changing mRNA abundance, dynamics, and primary and secondary structure [61-66]. Second, the microRNAs may be affecting translation rate but not transcript abundance, in which case their effects would not be detectable by microarray analysis. Finally, many of the microRNAs investigated likely regulate too few genes to be considered significant by this whole-genome target analysis, since a small list of targets can lead to artificially low statistical significance by bootstrap analysis. Indeed, some microRNAs might regulate a small number of critical genes and thereby produce an important functional effect even without a statistically significant change in the average proliferation index for all of its targets. For these reasons, we chose to investigate further *miR-29 *and other candidates identified based on their previously reported associations with proliferation and cell cycle regulation: *let-7 *[35,36] and *miR-125 *[32,33].

### *miR-29 *regulates collagen and collagen-chaperone genes

**Figure 3:**
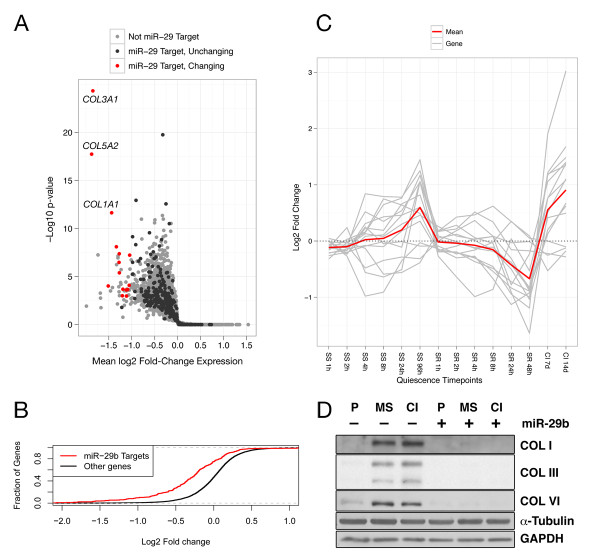
***miR-29 *repression of extracellular matrix protein production with quiescence**. (**A**) Gene expression changes induced 48 h after *miR-29 *transfection into fibroblasts. The *x*-axis denotes the mean log_2 _fold change in expression compared to negative control, and the y-axis denotes -log_10 _of the *P *value of a one-sided *t*-test. (**B**) Empirical cumulative distribution function of log_2 _fold-changes induced by *miR-29 *transfection, comparing predicted targets to all other non-target genes. (**C**) Quiescence microarray expression timecourses (Figure 2A) of each *miR-29 *target in Table 1 (shown in gray), along with the mean log_2 _fold change at each timepoint (shown in red). (**D**) Protein expression, as determined by immunoblotting, of selected *miR-29 *targets in proliferating (P), mitogen-starved (MS), or contact inhibited (CI) states with transfection of a negative control microRNA or *miR-29*. Collagen III here appears as a doublet corresponding to its two isomers. Immunoblots to GAPDH and α-Tubulin are shown as examples of genes not targeted by miR-29 and as loading controls.

Gene ontology analysis of predicted, evolutionarily conserved *miR-29 *targets revealed an enrichment for multiple categories including collagen fibril organization and extracellular matrix formation (Additional File 1, Table S3), indicating that *miR-29 *most likely regulates extracellular matrix (ECM) biosynthesis in fibroblasts, consistent with previous reports on *miR-29 *in fibroblasts and other cell types [67-72]. We identified *miR-29 *targets in dermal fibroblasts by overexpressing *miR-29 *in asynchronously proliferating fibroblasts and analyzing the ensuing changes in gene expression by microarray analysis. As expected, genes predicted to be *miR-29 *targets by TargetScan were more likely to be repressed by *miR-29 *overexpression than genes not predicted to be *miR-29 *targets (Figure 3B). We identified genes that both changed significantly in the microarray analysis and contained predicted *miR-29 *binding sites. Of the 15 genes that met these criteria, nine are involved in extracellular matrix formation (Figure 3A and Table 1). When we plotted the behavior of these same genes in the serum starvation and contact inhibition microarray timecourse data, we discovered that these genes display a quiescence-associated gene expression pattern. The genes encoding *miR-29 *targets followed a general pattern of increasing expression as fibroblasts are serum-starved, decreasing expression as they are restimulated, and highest expression in cells that were contact-inhibited for 7 or 14 days (Figure 3C). These genes were therefore highly anti-correlated with the pattern of expression for *miR-29 *itself (Additional File 1, Figure S4). These results suggest that the downregulation of *miR-29 *expression levels in quiescent fibroblasts is an important contributor to the induction of extracellular matrix genes with quiescence.

**Table 1:** *miR-29 *experimentally-determined targets.

Gene	Log_2 _fold change	Function
*ARRDC4*	-1.19	N/A

*BLMH*	-1.05	N/A

*CDK6*	-1.27	Cell cycle

*COL1A1*	-1.44	ECM

*COL3A1*	-1.85	ECM

*COL5A2*	-1.87	ECM

*FBN1*	-1.27	ECM

*FSTL1*	-1.51	BMP antag.

*LAMC1*	-1.06	ECM

*MFAP2*	-1.11	ECM

*PPIC*	-1.28	ECM?

*RCC2*	-1.21	Cell cycle

*SERPINH1*	-1.09	ECM

*SPARC*	-1.34	ECM

*TBC1D7*	-1.12	N/A

Genes listed were significantly repressed by *miR-29 *transfection according to a one-sided *t*-test at 5% FDR, had log_2 _fold changes of <-1.0, and are evolutionarily conserved *miR-29 *targets as annotated by TargetScan.

We sought to confirm whether *miR-29 *regulates not just transcript abundance, but also protein levels of extracellular matrix components in quiescent cells. We investigated three proteins encoded by *miR-29 *targets (collagen I, collagen III, and collagen VI) by immunoblot analysis of protein lysates isolated from proliferating cells and cells made quiescent by mitogen (PDGF) withdrawal or contact inhibition. As anticipated, all three proteins were upregulated in both quiescence conditions compared with proliferating cells. These three *miR-29 *targets were also strongly repressed at the protein level by transfection of *miR-29 *as compared to transfection of a negative control, non-targeting microRNA, while protein levels of GAPDH and α-tubulin (two proteins from genes not targeted by *miR-29*) were unaffected (Figure 3D).

### Autocrine TGF-ß is unlikely to mediate *miR-29 *expression changes in quiescence

TGF-ß signaling leads to an increase in collagen synthesis [73] and can repress *miR-29 *[69,74,75]. We confirmed that exogenous addition of TGF-ß repressed *miR-29 *expression, as measured by qRT-PCR (Additional File 1, Figure S5A), in our dermal fibroblast model. Although exogenous TGF-ß can downregulate *miR-29*, immunoblots for Smad3 phosphorylation levels showed no significant difference in autocrine TGF-ß signaling between proliferating and quiescent fibroblasts (Additional File 1, Figure S5B), indicating that the TGF-ß signaling pathway is unlikely to be responsible for the reduction in *miR-29 *expression in quiescent fibroblasts. In addition, although TGF-ß can regulate collagen expression independently of *miR-29 *[76,77], the similar phospho-Smad3 levels in proliferating and quiescent fibroblasts implies that changes in TGF-ß activity are unlikely to significantly regulate collagen biosynthesis in quiescence, further emphasizing the importance of *miR-29 *as a regulator of quiescence-associated changes in ECM expression.

### *miR-29 *hastens cell cycle re-entry from quiescence

**Figure 4:**
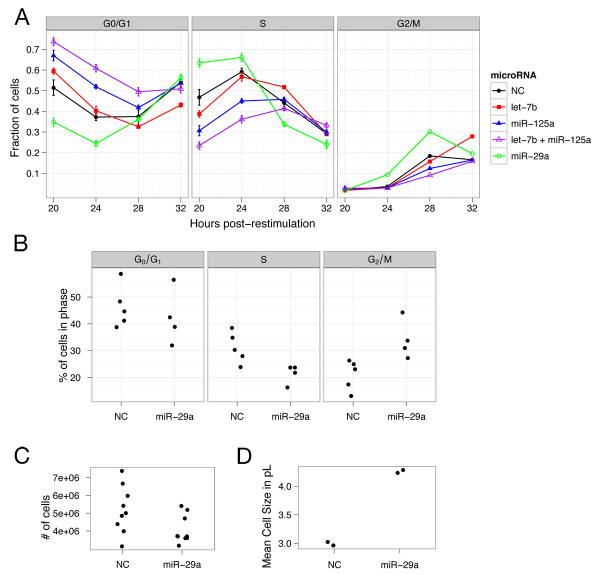
**Cell cycle and cell size effects of microRNAs *let-7*, *miR-125*, and *miR-29***. (**A**) Cell cycle progression of serum-restimulated quiescent cells with simultaneous transfection of *miR-29*, *let-7*, *miR-125*, a combination of *let-7 *and *miR-125*, or a negative control (NC) non-targeting miRNA. The fraction of cells in different cell cycle phases is plotted with error bars of the residual sums of squares from two timecourses measured in triplicate. (**B**) Cell cycle phase distribution of asynchronously proliferating fibroblasts 48 h after transfection with *miR-29*. (**C**) Cell numbers 48 h after *miR-29 *transfection. (**D**) Cell sizes 48 h after *miR-29 *transfection.

We also tested whether *miR-29 *has a role in the cell cycle transition between proliferation and quiescence by simultaneously restimulating serum-starved fibroblasts to proliferate with full serum medium and transfecting them with *miR-29*. Over the next 36 h, we quantified by flow cytometry the rate of EdU nucleotide analogue incorporation by the cells and their overall DNA content, which allowed us to assign cells to G_0_/G_1_, S, and G_2_/M phases of the cell cycle [78]. When compared to cells transfected with a control non-targeting microRNA, cells transfected with *miR-29 *contained fewer cells in G_0_/G_1 _and more cells in S phase at 20 and 24 h post transfection (Figure 4A, $P=1.9\times 10^{-7},3.0\times 10^{-11}$ for 20 and 24 h timepoints, respectively). At 28 and 32 h after transfection, cells transfected with *miR-29 *contained fewer cells in S phase and more cells in G_2_/M phase than those transfected with the control (P=0.012 for 28 h timepoint). *miR-29 *overexpression thus hastens re-entry into the cell cycle from a quiescent state.

To further explore the effects of *miR-29 *expression on the cell cycle, we transfected *miR-29 *or a negative control microRNA into asynchronously cycling fibroblasts. Forty-eight hours post transfection, *miR-29 *transfection led to more cells in G_2_/M (Figure 4B). As expected considering that cells in the G2/M phase tend to be larger than cells in other phases of the cell cycle, *miR-29 *transfection also led to larger cells (Figure 4D). Further experimentation revealed that *miR-29 *transfection resulted in fewer cells than the negative control transfection (Figure 4C, P=0.025). Thus, *miR-29 *transfection in proliferating cells led to G_2_/M arrest rather than increased mitosis. This may reflect the activity of a *miR-29 *target gene; indeed, one target, *RCC*2 (*TD-60*), is repressed about 57% upon *miR-29 *transfection (Figure 3A and Table 1), and it plays an essential role in progression through metaphase [79].

### *let-7 *and *miR-125 *non-redundantly delay cell cycle entry from quiescence

*let-7 *plays roles in differentiation, cancer, and the cell cycle, as discussed above. In *C. elegens*, the *lin-4 *microRNA (*miR-125 *in mammals) acts in the same heterochronic pathway of temporal differentiation as *let-7 *[27]. The two microRNAs are also frequently located together in microRNA clusters across many phylogenetic lineages [80]. In multiple species, they are co-regulated and share partly overlapping roles during development [81-86]. Because both *let-7 *and *miR-125 *are upregulated in quiescence, we investigated whether *let-7 *and *miR-125 *have complementary roles in cell cycle regulation.

We monitored the functional roles of *let-7 *and *miR-125 *on cell cycle re-entry from quiescence using the same method we used for *miR-29 *as described above. Compared with control-transfected cells, cells transfected with *let-7 *contained an elevated fraction of cells in the G_0_/G_1 _phase at 20 and 24 h post transfection and fewer cells in S phase at 20 h post transfection (Figure 4A, P=0.0042,0.0083 for 20 and 24 h timepoints, respectively), indicating that cell cycle re-entry is delayed by *let-7 *overexpression. By 32 h post transfection, the *let-7*-overexpressing population contained more cells in the G_2_/M phase than control cells (P=0.0013), as we have previously reported [36]. We observed an even stronger effect on cell cycle re-entry with *miR-125 *than for *let-7*. At 20 and 24 h after transfection, cells transfected with *miR-125 *contained more cells in G_0_/G_1 _and fewer cells in S phase than controls (Figure 4A, $P=7.5\times 10^{-6},6.0\times 10^{-9}$ for 20 and 24 h timepoints, respectively). To assess whether *let-7 *and *miR-125 *have complementary effects on cell cycle progression, we overexpressed a combination of the two microRNAs. Overexpression of *let-7 *and *miR-125 *together resulted in a further accumulation of cells in G_0_/G_1 _and even slower S phase entry than either individually (Figure 4A, $P=1.0\times 10^{-8},1.0\times 10^{-4}$ compared to *let-7 *and *miR-125*, respectively, at the 20 h timepoint), implying that their cell cycle effects are non-redundant and complementary.

## Discussion

### A microRNA quiescence program

While the predominant view of quiescent cells is that they are inactive or 'shut down', our data from several different lines of experimentation indicate that the transition into quiescence in fibroblasts is a highly regulated and active process [2,14,52]. We previously reported [2], and we again confirmed by our SVD analysis of quiescence gene expression timecourse data (Figure 2), that entry into quiescence in fibroblasts is associated with large-scale remodeling of gene expression patterns affecting a significant fraction of all genes within the genome, with comparable numbers of genes both increasing and decreasing in expression. We show here that entry into quiescence is also associated with widespread changes in the abundance of a significant number of microRNAs. microRNAs both increase and decrease in abundance upon entry into quiescence, similar to the effects on mRNA expression.

One clear distinction between microRNAs and mRNAs was noticed: while gene expression patterns have both a common component and a signal-specific component [2] (Figure 2A), microRNA patterns with quiescence were very similar for samples made quiescent by two distinct quiescence signals (contact inhibition and serum starvation). This finding is in accord with previous studies that indicated that microRNA profiles are extremely informative about a human cancer's developmental lineage and differentiation state, and that microRNAs are particularly valuable for classifying poorly differentiated tumors [87,88]. Indeed, our data suggest that there may be a quiescence microRNA program that is stronger and more consistent than a quiescence gene expression program. Such a signature may facilitate the identification of universal quiescence-related pathways.

### The complementarity of *let-7 *and *miR-125*

In many organisms, *lin-4 *(*miR-125*) and *let-7 *are both important for developmental programs involving differentiation or cell cycle arrest [26,31]. Low levels of *let-7*, for example, are associated with pluripotency and proliferation, while higher *let-7 *levels are associated with cell cycle exit and differentiation [28,34,89]. In vertebrates, mature *let-7 *and *miR-125 *are largely absent from early embryos and are induced upon differentiation [84-86]. We previously reported that *let-7 *targets the E2 ubiquitin ligase *CDC34 *and that *let-7 *overexpression in fibroblasts results in a G_2_/M arrest [36]. Here we show that, when overexpressed, both *miR-125 *and *let-7 *specifically affect the ability of quiescent fibroblasts to re-enter the proliferative cell cycle from quiescence induced by serum starvation.

Our data and the literature, taken together, support a model in which *miR-125 *and *let-7 *family members are induced upon the commitment to a cell state lineage or reversible cell cycle exit. During differentiation or quiescence, *let-7 *and *miR-125 *may actively suppress the expression of cell cycle-associated transcripts through a post-transcriptional mechanism that reinforces the out-of-cycle state established by transcriptional mechanisms. Possible candidates for these transcripts include previously reported cell cycle targets of *let-7 *such as *RAS *[39], *CCND1 *[90], *CDC25 *[35], and *CDC34 *[36], and *miR-125 *targets such as *BCL3 *[91] and *ETS1 *[92]. Our results indicate that in reversibly arrested cells, *miR-125 *and *let-7 *downregulate cell proliferation-promoting genes. Upon restimulation, these genes are released from *let-7 *and *miR-125*-mediated repression and are required for normal cell cycle re-entry.

Although *miR-125 *and *let-7 *are co-conserved and co-regulated in many organisms, the two microRNAs also share some overlapping target genes [33,93,94], which suggests the possibility that some of the functional effects on the cell cycle exerted by each microRNA are redundant. Our results demonstrate that introduction of both microRNAs together had a stronger effect on cell cycle re-entry than introduction of either one alone, suggesting that they cooperate and play non-redundant roles in suppressing the expression of proliferation-associated genes in quiescent cells. This finding helps to explain the strong evolutionary selection to retain both microRNAs. Exogenous delivery of the *let-7 *microRNA has been shown to cause regression of murine lung tumors through an effect on cell proliferation distinct from apoptosis [45]. Our data indicate that administration of *miR-125 *or a combination of *let-7 *and *miR-125 *might have even greater effects.

### *miR-29*'s role in quiescence

One of the functional changes that we previously observed in quiescent fibroblasts is an overall induction of extracellular matrix proteins [52]. We report here that downregulation of the microRNA *miR-29 *is likely regulating the induction of extracellular matrix protein expression with quiescence: as *miR-29 *levels decline with quiescence, levels of *miR-29 *targets increase, and *miR-29 *overexpression represses the levels of these targets. Reporter assays by multiple independent groups have found in several different cell types that *miR-29 *directly targets collagens *COL1A1*, *COL3A1*, and *COL4A2 *in a seed sequence-dependent manner [95-97]. Based on those studies and our microarray and immunoblot results, *miR-29 *likely also represses collagens directly in proliferating fibroblasts. The findings place *miR-29 *among the very few molecules discovered, along with FoxO [98-100], and FoxP [101,102] transcription factors, and the regulators of *miR-29 *itself, to regulate the induction (as opposed to the repression) of genes in quiescent cells. Because our data indicate that the activity of the TGF-ß signaling pathway is similar in proliferating and quiescent fibroblasts, it is not likely that TGF-ß is regulating the changes in *miR-29 *expression between these states. Other possible candidates for *miR-29 *transcriptional regulation include NF-κB and sonic hedgehog [70,103]. Further study is necessary to elucidate which factors are responsible in quiescence.

Repression of *RCC2 *could explain the G_2_/M arrest phenotype seen with *miR-29 *transfection. Targets identified in other model systems could also be relevant. *miR-29 *targeting of DNA methyltransferases 3A and 3B, for example, can inhibit lung cancer cell tumorigenicity [104]. *miR-29 *can also induce apoptosis in cholangiocarcioma cells via the *miR-29 *target *MCL-1 *[105], and induce replicative senescence in HeLa cells by targeting *B-MYB *[106].

We suggest that the role of *miR-29 *in hastening cell cycle re-entry, however, may reflect its effects not on validated cell cycle regulators, but instead on extracellular matrix proteins. Quiescent cells, in general, are relieved of the biosynthetic requirement of synthesizing the constituents of new cells, but in our fibroblast model system they also retain a comparable rate of metabolic activity as proliferating fibroblasts [52]. Indeed, we discovered that fibroblasts express increased levels of several extracellular matrix proteins during quiescence compared with proliferation [52] (Figures 3C and 3D). From this perspective, it is particularly interesting that *miR-29 *overexpression results in more rapid cell cycle entry. Although *miR-29 *has been reported to be an oncogene (transgenic mice overexpressing *miR-29 *in their B cells develop B-cell chronic lymphocytic leukemia [107]) our microarray data revealed no clear candidate cell cycle genes that would explain the early re-entry phenotype we observed in our model system.

We suggest an alternative possibility: relieved of the commitment to translate and fold extracellular matrix proteins like collagen, *miR-29*-overexpressing cells may be able to commit more rapidly to the cell cycle. If a competition exists for translational resources between the synthesis of proteins required for cell duplication and the synthesis of proteins targeted for secretory pathways, then *miR-29 *may be able to direct resources between those two processes depending on the proliferative state of the cell. Further studies, especially on fibroblast cell lines derived from patients with idiopathic pulmonary fibrosis, which are characterized by excessive secretion of extracellular matrix proteins [108,109], will be able to elucidate whether *miR-29 *is an important regulator of a tradeoff between proliferative and secretory modes.

## Conclusions

Our data indicate that quiescence is associated with widespread, consistent changes in microRNA abundance. The regulated microRNAs contribute to gene expression programs that form the characteristic attributes of quiescent cells by reinforcing the non-proliferative nature of the cells and also regulating their cell-type specific roles. As such, further investigation into microRNAs should lead to a greater understanding of both universal aspects of quiescence programs as well as the regulation of processes specific to a quiescent cell's *in vivo *roles. Our results support some of the ongoing efforts to administer microRNAs to patients of cancer and fibrotic disease and suggest some new strategies.

## Materials and methods

### Cell culture

We isolated primary fibroblasts from neonatal human foreskin tissue samples provided by the National Disease Research Interchange (NDRI) as described in the supplementary methods for Legesse-Miller *et al. *[36] We routinely cultured the fibroblasts aseptically at 37°C with 5% CO_2 _in high-glucose DMEM with 4.5 mM glutamine (Life Technologies) supplemented with 10% (v/v) fetal bovine serum (FBS) (Hyclone) and 100 μg/mL penicillin and streptomycin (Life Technologies). Cells were serum-starved by reducing the serum concentration to 0.1% (v/v). To generate contact-inhibited samples, we plated fibroblasts and changed their culture medium regularly (every 2 or 3 days) without passaging them.

### microRNA microarrays

Three isolates of dermal fibroblasts were harvested in proliferative conditions, that is, sparsely subcultured 2 days before harvest, after 4 days of serum starvation, or after 7 days of contact inhibition. Cells were harvested by trypsinization, centrifuged at 160 × *g*, and snap-frozen in liquid nitrogen. Total RNA was isolated from the frozen cells using the mirVana miRNA isolation kit (Life Technologies). RNA quality was confirmed using a Bioanalyzer 2100 (Agilent Technology) and the concentration was determined with a NanoDrop spectrophotometer (NanoDrop Technologies). 100 ng of each sample was 3′-labeled with Cy3-pCp in two separate reactions and hybridized to microarray slides using the Agilent microRNA microarray kit (Agilent, G4470A). Microarray features were extracted with Feature Extractor 9.5.3.1. We normalized arrays for total intensity and then regressed each gene's expression using the model

$$Y_{i}=m_{i}+B_{i,Q}x_{Q}+B_{i,s}x_{s}+B_{i,c_{1}}x_{c_{1}}+B_{i,c_{2}}x_{c_{2}}+B_{i,\text{SVA}}x_{\text{SVA}}+E_{i,Q,s,c_{1},c_{2},\text{SVA}},$$

where *i *denotes the index for a microRNA, *Q, S, C_1_*, and *C_2 _*are annotations for quiescence, serum starvation, and the different fibroblast cell isolates, respectively, and *SVA *denotes the one significant surrogate variable we found as described below. *Y_i _*is the measured log_2 _expression for microRNA *i *and *m_i _*is its baseline expression. The *x *variables are the given experimental variables (indexed by subscripts) with values 0 or 1, the *B *coefficients are the gene-specific responses to a particular *x *variable, and *E *is the error term. Surrogate variable analysis (SVA) was performed with the R package from Leek *et al. *[110], giving the one significant surrogate variable we included in the multiple regression analysis. Differential expression due to quiescence was determined with an *F*-test for the significance of the microRNA's response to variable *x_Q_*, with a false-discovery rate of 1% deemed statistically significant. microRNAs without statistically significant gene expression change from quiescence were not shown in Figure 1A and 1B.

We denoted the overall biological response to serum starvation and contact inhibition (plotted log_2 _transformed in both Figure 1A as the heat-map intensities and Figure 1B along the *x*-axis) as the sum of the responses $B_{i,Q},B_{i,S}$ and the residuals $E_{i,Q,S,C_{1},C_{2},\text{SVA}}$. The Pearson correlation coefficient was calculated comparing these values in the serum starvation and contact inhibition conditions.

### Multiplexed real-time PCR for microRNA expression levels

We collected primary human fibroblasts over a timecourse during serum starvation. Copy number of each microRNA per 10 pg of total RNA was determined using the protocol described in [53]. In summary, RNA was extracted using the mirVana microRNA isolation kit as described above, and a tailed, gene-specific primer was used to convert the RNA template into cDNA with a universal PCR binding site at one end. The resulting primer-extended, full-length cDNA was amplified in a highly multiplexed manner for 219 individual microRNAs. Real-time PCR was performed with a combination of an LNA-containing microRNA/siRNA-specific 'reverse' primer and a generic universal primer complementary to the universal binding site introduced during reverse transcription. Amplification was monitored with SYBR green fluorescence. The cycle number at which the signal exceeded the background was used to determine the absolute abundance of the monitored microRNA in the sample. The Pearson's correlation between the real-time PCR data and the microRNA microarray data was determined between the 4-day serum-starved data point for the qRT-PCR and the mean of the $B_{i,S}$ serum starvation responses from the multiple regression for the microarray.

### Gene expression microarrays for quiescence and *mir-29 *targets

Contact-inhibited fibroblast gene expression microarrays and serum starvation and restimulation arrays have been previous described [52,54]. To summarize briefly, total RNA was isolated from proliferating, serum-starved, and serum-stimulated fibroblasts as described above for the microRNA microarray. Total RNA from each sample, 325 ng each, was amplified and labeled using the Low RNA Input Fluorescent Labeling Kit (Agilent Technologies) to incorporate Cyanine 3-CTP (Cy-3) or Cyanine 5-CTP (Cy-5). Cy-3-labeled time zero samples were used as a reference for serum withdrawal samples, which were labeled with Cyanine 5-CTP. For serum stimulation, 4-day serum-starved fibroblasts were labeled with Cy-3 and stimulated samples were labeled with Cy-5. Labeled cRNA was mixed and co-hybridized to whole Human Genome Oligo Microarray slides (Agilent Technologies) at 60°C for 17 h and subsequently washed with the Agilent Oligo Microarray Hybridization Kit. Slides were scanned with a dual laser scanner (Agilent Technologies). The Agilent feature extraction software, in conjunction with the Princeton University Microarray database, was used to compute the log ratio of the difference between the two samples for each gene after background subtraction and dye normalization. Of the approximately 44,000 probes on the microarray, probes that generated signal in at least 80% of arrays were identified. Fluorescence data for each probe were mapped to genes based on UniGene Clusters. If multiple probes mapped to a single gene, the values were averaged. The Pearson correlation coefficient was computed between the 96h serum-starved sample and the mean of the 7d contact-inhibited samples. Hierarchical clustering was performed on centered genes via centroid linkage, and four clusters were chosen based on the resulting dendrogram. Gene ontology (GO) term enrichment was determined using the Generic Gene Ontology Term Finder [111]. Qualitatively non-informative or redundant GO terms (for example, 'Biological process' or 'cell cycle' *vs*. 'cell cycle process') were removed to give a selected subset.

For *miR-29 *overexpression microarrays, fibroblasts were transfected as described below with Pre-miR *miR-29b *or Negative Control #2 oligonucleotide duplexes (Life Technologies). Forty-eight hours after transfection, total RNA from the cells was harvested and hybridized to microarrays as above. The experiments were repeated on three different dermal fibroblast isolates. Target genes annotated by TargetScan 5.1 [55,56,112] were considered well-conserved *miR-29 *targets if P_CT _>0.5. A one-sided *t*-test was used to calculate the significance of the log_2 _fold change between the *miR-29b *transfection and the control, and a gene was declared 'changing' if it was repressed greater than two-fold at 5% FDR.

### Singular value decomposition to identify microRNAs with significantly changing predicted targets

The matrix of gene expression arrays was filtered to exclude genes with missing values in any array, and this matrix was decomposed by singular value decomposition (SVD) to obtain 16 eigengenes. Each gene's expression profile was then linearly projected onto the first eigengene to obtain one summarizing number, dubbed the 'proliferation index', as genes with a strong positive projection tend to be associated with proliferation and genes with a strong negative projection tend to be associated with quiescence. Sets of computationally-predicted target genes were obtained from TargetScan by excluding all predictions with context scores >-0.5 (negative numbers indicate more confident predictions). The mean projection of each of these target gene sets and its additive inverse were used as two-tailed test statistics on a null hypothesis distribution of 10,000 mean projections of randomly sampled gene sets. Each sample gene set was the same size as the original target gene set for which the linear projection was calculated.

### Overexpression of microRNA mimics

Proliferating or 4-day serum-starved primary fibroblasts were reverse-transfected using Oligofectamine (Life Technologies) with a 50 nM final concentration of Pre-miR microRNA duplexes *let-7b*, *miR-125a*, *miR-29a*, a 1:1 combination of *let-7b *and *miR-125a*, or the Negative Control #2 non-targeting control (Life Technologies). The microRNA duplexes and Oligofectamine were diluted in OptiMEM I (Life Technologies) and incubated at room temperature for 15 min. Human fibroblasts were trypsinized, washed, and then re-suspended in OptiMEM I at a concentration of 375,000 cells/mL. One milliliter of the transfection mixture was added to 4 mL of the cell suspension and plated on a 10 cm plate. The cells were incubated for 4 h and then supplemented with 5 mL of DMEM with 20% FBS. Twenty-four hours post transfection the medium was changed to DMEM containing 10% FBS.

For the serum-restimulation timecourses, we measured the duration of serum restimulation from the moment at which DMEM with 20% FBS was added. These experiments were done in triplicate on two different days (six timecourses in total). Standard error was calculated for both G_0_/G_1 _and S phase percentages at each timepoint as the square root of the total sum of square residuals from the mean percentage on each day. Proliferating cells were harvested 48 h after transfection for the assays described below.

### Cell cycle progression assay

We determined cell cycle phases using Click-iT EdU Alexa Fluor 488 according to the protocol in [78]. Briefly, we added 10 μL of a 10 mM EdU solution (Life Technologies) in phosphate-buffered saline (PBS) (Life Technologies) directly to 10 mL of culture medium on fibroblasts for a final concentration of 10 μM. We incubated the cells for 2 h with the EdU, and then trypsinized and re-suspended them to 1 × 10^7 ^cells/mL in PBS containing 1% bovine serum albumin (BSA) (Amresco). A total of 100 μL of this cell suspension was added to 100 μL of freshly prepared 4% formaldehyde in PBS (Thermo Scientific) and incubated in the dark at room temperature for 15 min. Three milliliters of PBS with 1% BSA was added to quench the fixation. The cells were then resuspended in 100 μL of PBS containing 1% BSA and added to 100 μL of 0.2% Triton X-100 in PBS. We added to each sample 500 μL of Click-iT reaction cocktail: 100 mM Tris-Cl, pH 8.5, 2 mM CuSO_4_, 10 μM Alexa Fluor 488 azide (Life Technologies), and 100 mM ascorbic acid. The mixture was incubated in the dark at room temperature for 30 min. Two milliliters of wash buffer (1% BSA, 0.2% Triton X-100 in PBS) was added, the cells were pelleted at 200 × g for 5 min, and the supernatant was discarded. We then resuspended the labeled cells in 500 μL of DAPI solution containing 1 μg/mL of DAPI in 0.1% Triton X-100 in PBS and analyzed them by flow cytometry on an LSR II flow cytometer (BD Biosciences, San Jose, CA, USA). DAPI was excited at 345 nm and its emission was detected at 458 nm. Alexa Fluor 488 was excited at 494 nm and its emission was detected at 519 nm.

Statistical significance of the changes was determined using a Dirichlet likelihood ratio test that is similar to a χ2 test of independence. To summarize, the cell cycle phase proportions at each timepoint for each microRNA transfection were fit to a maximum likelihood Dirichlet distribution by an iterated, alternating mean/precision estimation method [113]. The distributions and their log likelihoods were calculated for the null hypothesis of identical Dirichlet distributions and the alternative hypothesis of two different Dirichlet distributions for the negative control transfection and the microRNA transfection of interest. The log likelihoods of the two hypotheses were compared using the test statistic

$$D=-2\text{log}L\left(H_{o}|C\right)+2\text{log}L\left(H_{a}|C\right),$$

where *C *is the flow cytometry data at the particular timepoint. *D *was then evaluated on the χ2 distribution for three degrees of freedom to calculate a *P *value (the alternative hypothesis calculates two three-parameter Dirichlet distributions instead of one).

### Cell size and number analysis

Dermal fibroblasts were trypsinized and resuspended in PBS, and cell size was measured in triplicate for each sample using the Beckman Coulter counter. Cell numbers were determined using the Countess automated cell counter (Invitrogen). For *miR-29 *transfection, cell numbers were evaluated using a one-sided *t *test.

### Immunoblotting for *miR-29 *targets

Fibroblasts were reverse transfected with *miR-29b *or a negative control microRNA as above, but cells were plated at either 7,500 cells/cm^2 ^(proliferating and mitogen-starved conditions) or 750,000 cells/cm^2 ^(contact-inhibited condition). Twenty-four hours post transfection, cells were washed with warm PBS and then switched to low-serum conditions for collecting extracellular matrix proteins: FBM (Lonza), insulin (Lonza), and 0.1% FBS (v/v). Proliferating and contact-inhibited conditions were additionally supplemented with 30 ng/mL recombinant human PDGF-BB.

After culturing for 4 days in low-serum medium, intracellular proteins were collected by washing cells in ice-cold PBS followed by scraping cells into a solution of 4% SDS, 100 mM Tris-HCl pH 7.5, 1 mM DTT, and an EDTA-free protease inhibitor cocktail (Roche). Lysates were vortexed, heated briefly (5-10 min at 95°C), sonicated until the solutions became non-viscous, and then centrifuged at 10,000 × g for 10 min. The soluble lysates were transferred to new tubes and insoluble pellets were discarded. Determination of soluble lysate concentration and immunoblotting conditions were otherwise identical to those previously described in Lemons *et al. *and Pollina *et al. *[52,54]. Antibodies and specific blotting conditions used are described below.

### TGF-ß treatment and signaling analysis

Cells were treated with recombinant human TGF-ß1 (Life Technologies) for 48 h in low-serum medium: high glucose DMEM with 4.5 mM glutamine, serum replacement (Sigma-Aldrich, S2640), and 30 ng/mL PDGF-BB. Cells were lysed in TRIzol, and RNA was harvested with the Direct-zol RNA Miniprep Kit (Zymo Research). The expression of *miR-29b *was measured in cell samples on a small scale normalized to *miR-100 *using the miRCURY LNA Universal RT microRNA PCR kit (Exiqon) with *miR-29b *and *miR-100 *primer sets and an ABI 7900 real-time PCR system. Total RNA samples were extracted using TRIzol reagent (Life Technologies) along with the Direct-zol RNA Miniprep kit (Zymo Research). Relative expression changes were quantified in triplicate using the $\Delta \Delta C_{t}$ method on SYBR green fluorescence. Cell lysates from proliferating, 4 days serum-starved, and 7 days contact-inhibited fibroblasts were harvested according to the procedures above.

### Antibodies

The following primary antibodies were used for immunoblotting: rabbit polyclonal IgG against collagen I (Calbiochem, 234167), rabbit polyclonal IgG against COL3A1 (Santa Cruz Biotechnology, sc-28888), biotinylated rabbit polyclonal IgG against Collagen VI (Acris Antibodies, R1043B), rabbit monoclonal IgG against Phospho-Smad3 Ser423/425 (Cell Signaling Technology, 9520), rabbit monoclonal IgG against α-Tubulin (Cell Signaling Technology, 2125), and rabbit polyclonal IgG against GAPDH (Abcam, ab9485). Each antibody was diluted in Tris-buffered saline containing 0.1% Tween-20 and 5% BSA and incubated with immunoblot membranes overnight at 4°C.

### Accession numbers

The microarray data generated for this study (the microRNA microarrays and the miR-29 overexpression microarrays) have been deposited in the NCBI Gene Expression Omnibus (GEO) [114] as one SuperSeries under the accession number GSE42614. Serum starvation/restimulation timecourse microarrays [54] and contact inhibition microarrays [52] were published in prior studies and are available in GEO with accessions GSE42681 and GSE42612, respectively.

## Abbreviations

CI: confidence interval or contact inhibition; EdU: 5-ethynyl-2'-deoxyuridine; FDR: false discovery rate; qRT-PCR: quantitative reverse-transcription polymerase chain reaction; SS: serum starvation.

## Competing interests

The authors declare that they have no competing interests.

## Authors' contributions

EJS, ALM, and JL conducted microarray experiments. EJS performed the statistical analyses and biochemical studies. EJ, MK, ALM, EJS, and MR conducted the molecular biology assays. ESS, TC, EJS, and MR conducted the cell cycle assays. EJS, ALM, JF, and HC conceived of the study, participated in its design and coordination, and helped to draft the manuscript. All authors read and approved the final manuscript.

## Supplementary Material

Additional file 1 **Contains additional tables and figures referred to in the text**. Click here for file
