# Supplementary material for: A microRNA network regulates proliferative timing and extracellular matrix synthesis during cellular quiescence in fibroblasts
Source: Genome Biol. 2012 Dec 22;13(12):R121. doi: 10.1186/gb-2012-13-12-r121 (PMC3924601; doi:10.1186/gb-2012-13-12-r121)

# Supplementary Tables

## Table S1. microRNAs that change in abundance in quiescent fibroblasts

microRNAs listed are those whose expression changes are statistically significantly influenced by quiescence (at FDR 0.1% with at least a 2-fold mean expression change contribution).

| **Greater in Proliferation** | **Greater in Quiescence** |
| --- | --- |
| miR-7 | miR-101 |
| miR-29b | miR-181a |
| miR-29b-1* | miR-199b-5p |
| miR-155 | miR-26a |
| miR-31* | miR-150* |
| miR-20a | miR-1225-5p |
| miR-93 | miR-874 |
| miR-17 | miR-940 |

## Table S2. Gene Ontology Terms for Quiescence mRNA microarrays

Select gene ontology terms for clusters obtained from hierarchical clustering of quiescence mRNA microarrays (as shown in Figure 2A).

| **Select GO Terms for Cluster I** |
| --- |
| Cell cycle |
| Mitosis |
| Ribosome biogenesis |
| ncRNA processing |
| RNA splicing |
| RNA localization |
| Exonucleolytic nuclear-transcribed mRNA catabolic process involved in deadenylation-dependent decay |
| Negative regulation of ubiquitin-protein ligase activity involved in mitotic cell cycle |
| Nuclear division |
| Organelle fission |
| Mitochondrion organization |
| Establishment of protein localization to mitochondrion |
| **Select GO Terms for Cluster II** |
| Cell cycle |
| Interphase of mitotic cell cycle |
| Protein ubiquitination |
| Exonucleolytic nuclear-transcribed mRNA catabolic process involved in deadenylation-dependent decay |
| Ribonucleoprotein complex biogenesis |
| RNA splicing |
| G2/M transition of mitotic cell cycle |
| Mitochondrion organization |
| Programmed cell death |
| **Select GO Terms for Cluster III** |
| No significant GO term enrichment. |
| **Select GO Terms for Cluster IV** |
| Collagen fibril organization |
| Protein ubiquitination |
| Plasma membrane organization |
| Positive regulation of intracellular protein kinase cascade |

## Table S3. Gene Ontology Terms for *miR-29* targets

Gene ontology terms for predicted targets of *miR-29* as given by TargetScan at < 2% false discovery rate.

| **GO Terms for miR-29 Targets** |
| --- |
| Collagen fibril organization |
| Chromosome organization |
| Transcription |
| Chromatin modification |
| Extracellular matrix organization |
| Vasculature development |
| Cell Cycle |
| Fibroblast proliferation |
| Platelet-derived growth factor receptor signaling pathway |
| Cell proliferation |
| Post-transcriptional regulation of gene expression |
| Cell motility |

# Supplementary Figures

## Figure S1. Comparison of correlation of mRNA and microRNA under different quiescence conditions

mRNAs and microRNAs, as measured by microarray, show different degrees of correlation between 4d serum starvation and 7d contact inhibition. On the *x*-axis is plotted the mean log_2_ fold-change in expression from proliferation to 4d serum starvation, and along the *y*-axis is plotted the mean log_2_ fold-change in expression from proliferation to 7d contact inhibition. Each point represents one microRNA or mRNA.


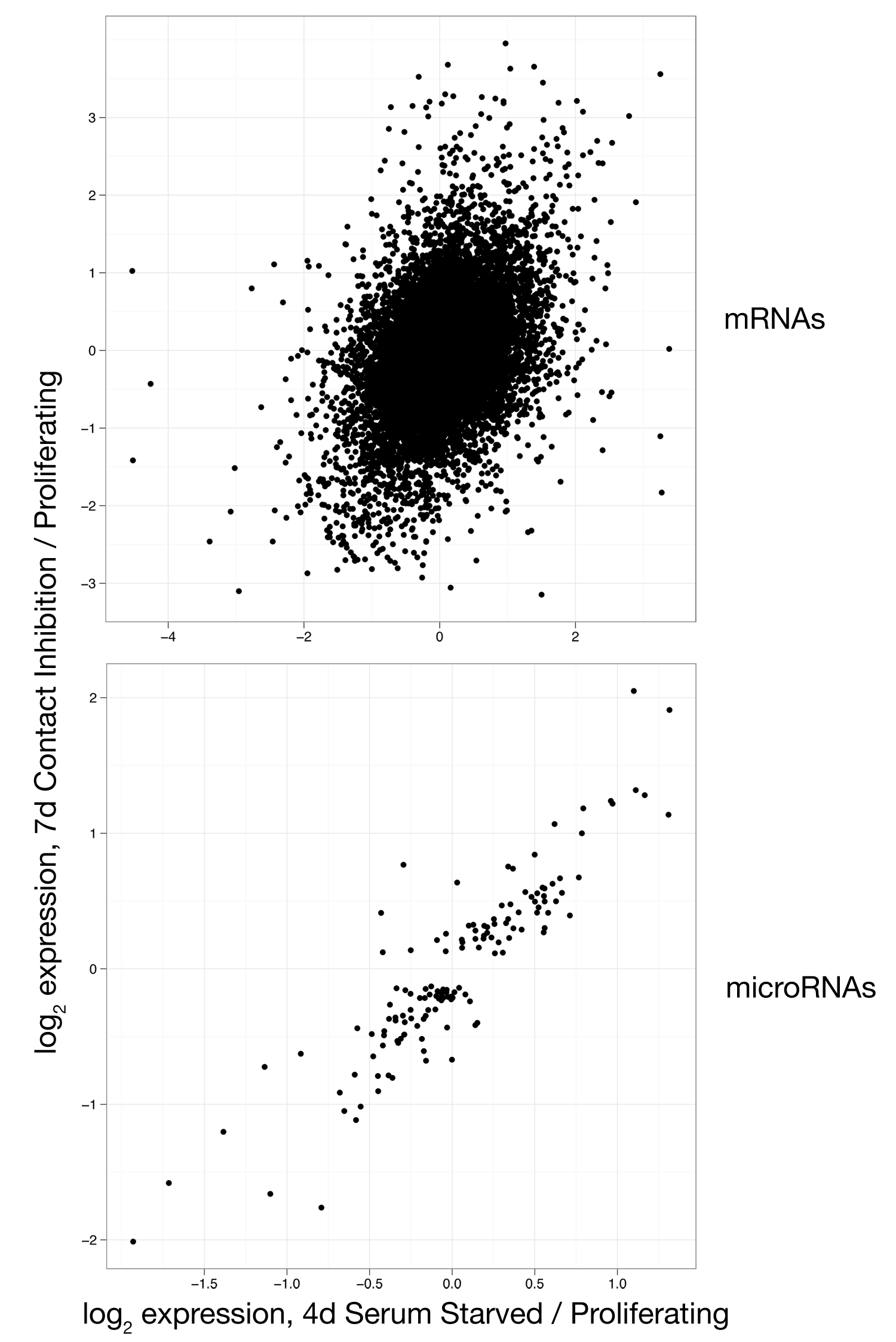


## Figure S2. Correlation of qPCR vs. microRNA microarray

High throughput qRT-PCR and microRNA microarray provide similar estimates of changes in microRNA abundance with quiescence. For each microRNA, the qRT-PCR measurement of its expression fold change (log_2_) from proliferation to 4 days serum starvation is plotted on the *y*-axis and its log_2_ mean fold change between proliferation and quiescence, as represented by the ANOVA quiescence coefficient $B_{i,Q}$ from the microRNA microarray, is plotted on the *x*-axis. The dotted line represents the ideal correlation $x=y$.


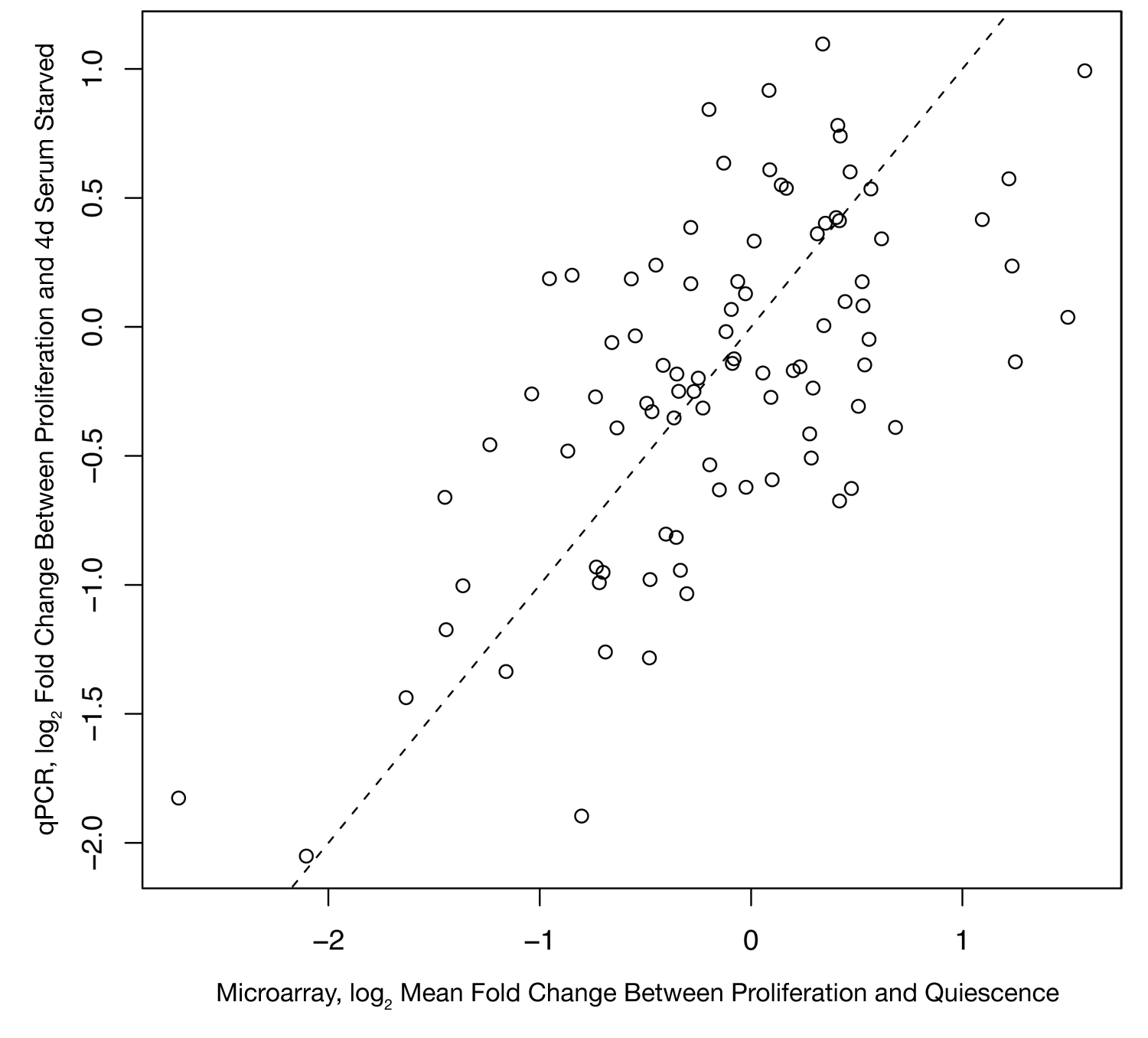


## Figure S3. Singular value decomposition of genome microarray

Singular value decomposition of the mRNA microarray captures most of the gene expression variation with two eigengene components. (A) microRNA microarray data was summarized by singular value decomposition and the percent of overall gene expression variation explained by each eigengene is plotted. (B) The gene expression profile of the leading eigengene, which explains about 38% of the gene expression variation in the microarray.


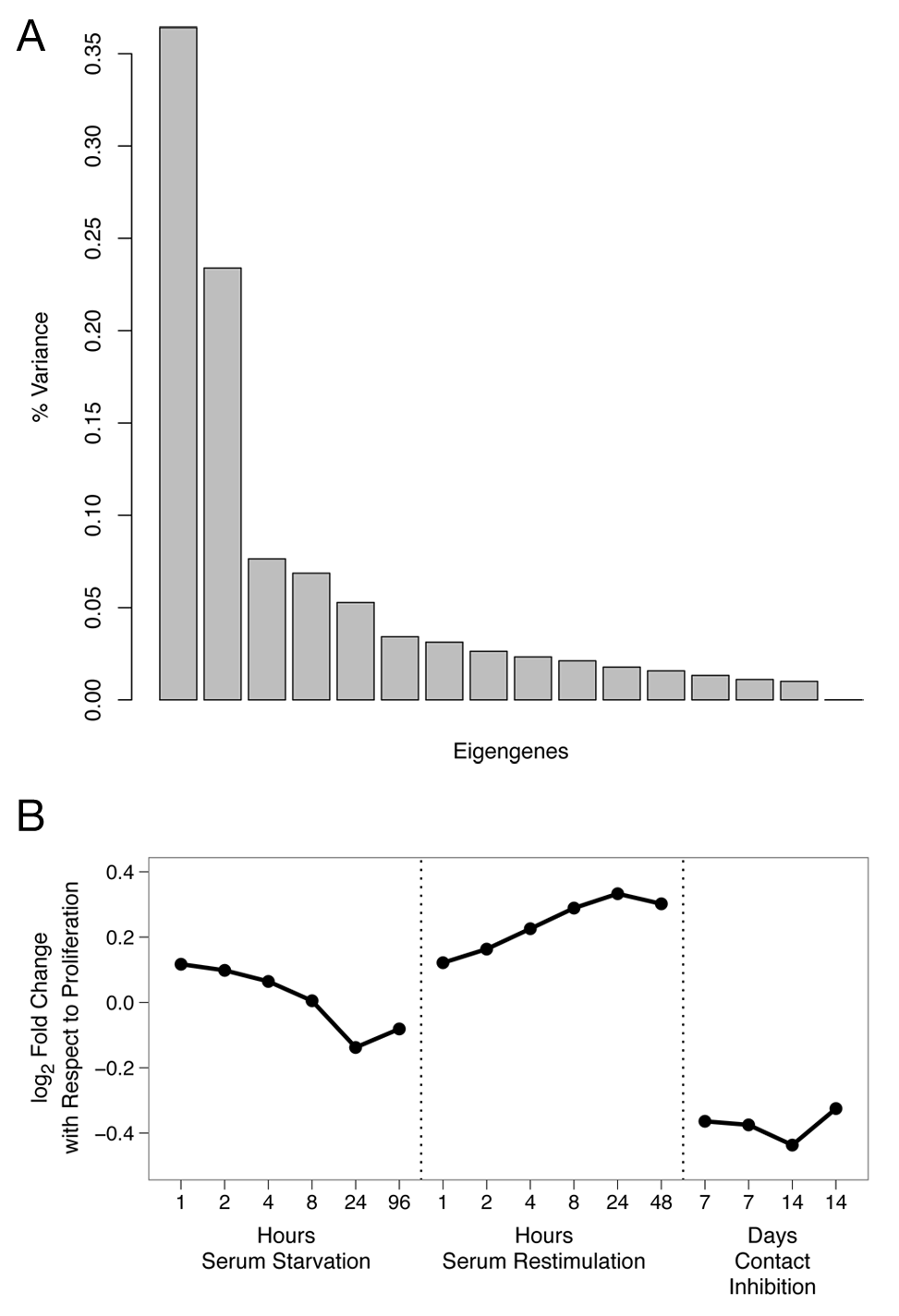


## Figure S4. *miR-29b* expression in microRNA microarrays

The expression of *miR-29b* from the microRNA microarrays shown in Figure 1A is plotted with log_2_ expression on the *y*-axis for each of the three cell cycle conditions. Two different probes detected miR-29b in each of two replicates from three cell isolates, giving 12 points for each condition.

## Figure S5. TGF-ß in proliferating and quiescent fibroblasts

TGF-ß can reduce miR-29 levels, but does not change during quiescence in these conditions. (A) Fibroblasts treated with different concentrations of TGF-ß were monitored for *miR-29* levels by qRT-PCR. (B) TGF-ß signaling levels in proliferating, 4 day serum-starved, and 7 day contact-inhibited fibroblasts were measured by blotting cell lysates with an antibody specific for Smad3 phosphorylation on serines 423 and 425. GAPDH was used as a loading control. A representative blot of two biological replicates is shown.


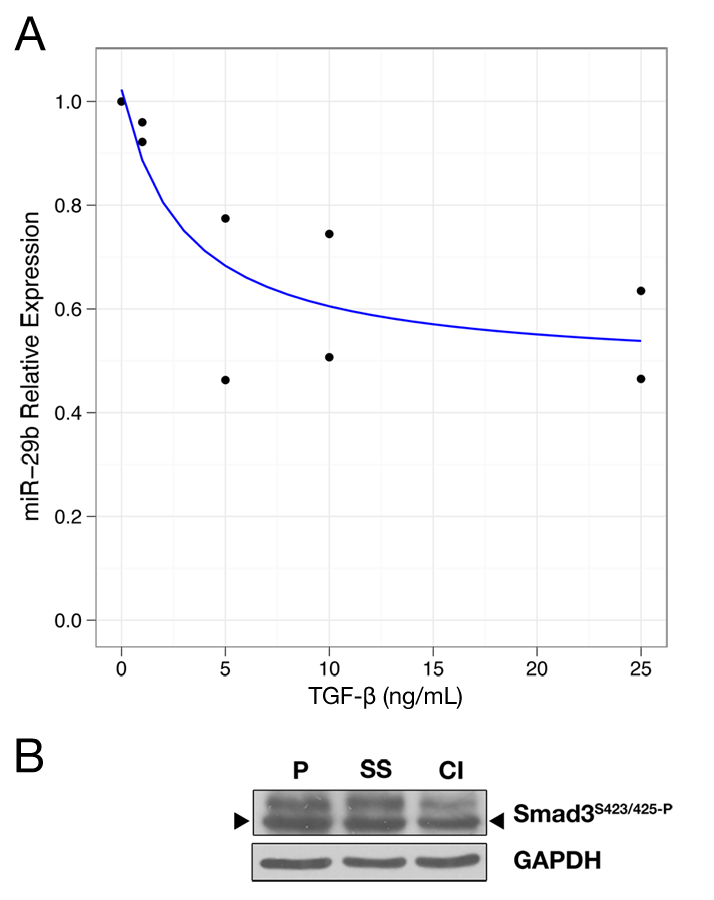

Supplement: Additional file 1 — Contains additional tables and figures referred to in the text. [file gb-2012-13-12-r121-S1.DOCX]
